# Toxicities in long‐term survivors of head and neck cancer—A multi‐national cross‐sectional analysis

**DOI:** 10.1002/ijc.70033

**Published:** 2025-07-30

**Authors:** Katherine J. Taylor, Cecilie D. Amdal, Kristin Bjordal, Guro L. Astrup, Bente B. Herlofson, Fréderic Duprez, Ricardo R. Gama, Alexandre Jacinto, Eva Hammerlid, Melissa Scricciolo, Femke Jansen, Irma M. Verdonck‐de Leeuw, Giuseppe Fanetti, Orlando Guntinas‐Lichius, Johanna Inhestern, Tatiana Dragan, Alexander Fabian, Andreas Boehm, Ulrike Wöhner, Naomi Kiyota, Maximilian Krüger, Pierluigi Bonomo, Monica Pinto, Sandra Nuyts, Joaquim Castro Silva, Carmen Stromberger, Pol Specenier, Francesco Tramacere, Ayman Bushnak, Pietro Perotti, Michaela Plath, Alberto Paderno, Noa Stempler, Maria Kouri, Vincent Grégoire, Silke Tribius, Susanne Singer, the EORTC Quality of Life Group and the EORTC Head and Neck Group

**Affiliations:** ^1^ Institute of Medical Biostatistics, Epidemiology, and Informatics University Medical Centre Mainz Mainz Germany; ^2^ Department of Oncology Oslo University Hospital Oslo Norway; ^3^ Research Support Service Oslo University Hospital Oslo Norway; ^4^ Faculty of Medicine University of Oslo Oslo Norway; ^5^ Faculty of Dentistry University of Oslo Oslo Norway; ^6^ Department of Otorhinolaryngology Oslo University Hospital Oslo Norway; ^7^ Department of Radiotherapy‐Oncology Ghent University Hospital, Faculty of Medicine and Health Sciences—Human Structure and Repair, Ghent University Ghent Belgium; ^8^ Department of Head and Neck Surgery Barretos Cancer Hospital SP Brazil; ^9^ Department of Radiation Oncology Barretos Cancer Hospital Barretos SP Brazil; ^10^ Department of Otorhinolaryngology‐Head and Neck Surgery Institute of Clinical Sciences, Sahlgrenska Academy at University of Gothenburg, Sahlgrenska University Hospital Gothenburg Sweden; ^11^ Department of Radiation Oncology Ospedale dell'Angelo Venice Italy; ^12^ Department Otolaryngology‐Head and Neck Surgery Amsterdam UMC location Vrije Universiteit Amsterdam the Netherlands; ^13^ Cancer Center Amsterdam Treatment and Quality of Life Amsterdam The Netherlands; ^14^ Department of Clinical, Neuro and Developmental Psychology Vrije Universiteit Amsterdam Amsterdam the Netherlands; ^15^ Division of Radiation Oncology Centro di Riferimento Oncologico di Aviano (CRO) IRCCS Aviano Italy; ^16^ Department of Otorhinolaryngology Jena University Hospital Jena Germany; ^17^ Department of Otorhinolaryngology Oberhavelkliniken Hennigsdorf Hennigsdorf Germany; ^18^ Department of Radiation Oncology, Head and Neck Unit Institut Jules Bordet, Université Libre de Bruxelles Brussels Belgium; ^19^ Department of Radiation Oncology University Hospital Schleswig‐Holstein Kiel Germany; ^20^ Department of Otorhinolaryngology St. George Hospital Leipzig Germany; ^21^ Cancer Center Kobe University Hospital Kobe Japan; ^22^ Department of Oral and Maxillofacial Surgery—Plastic Surgery University Medical Centre Mainz Germany; ^23^ Radiation Oncology Azienda Ospedaliero‐Universitaria Careggi Florence Italy; ^24^ Rehabilitation Medicine Unit Istituto Nazionale Tumori—IRCCS—Fondazione G. Pascale Naples Italy; ^25^ Laboratory of Experimental Radiotherapy, Department of Oncology KU Leuven Leuven Belgium; ^26^ Department of Radiation Oncology Leuven Cancer Institute, University Hospitals Leuven Leuven Belgium; ^27^ Department of Otolaryngology, Head and Neck Surgery Instituto Português de Oncologia Francisco Gentil Do Porto Porto Portugal; ^28^ Department of Radiation Oncology Charité‐Universitätsmedizin Berlin, corporate member of Freie Universität Berlin, Humboldt‐Universität zu Berlin Germany Berlin Germany; ^29^ Berlin Institute of Health Berlin Germany; ^30^ Department of Oncology Antwerp University Hospital Edegem Belgium; ^31^ Department of Radiation Oncology Azienda Sanitaria Locale Brindisi Italy; ^32^ Department of Otorhinolaryngology University Hospital Gießen und Marburg Giessen Germany; ^33^ Department of Otorhinolaryngology—Head and Neck Surgery “S. Chiara” Hospital, Azienda Provinciale Per I Servizi Sanitari (APSS) Trento Italy; ^34^ Department of Otorhinolaryngology, Head and Neck Surgery University Hospital Heidelberg Heidelberg Germany; ^35^ Department of Otorhinolaryngology—Head and Neck Surgery ASST Spedali Civili of Brescia, University of Brescia Brescia Italy; ^36^ Oral Medicine Unit Sheba Medical Center Tel Hashomer Israel; ^37^ Dental Oncology Unit, Department of Oral Medicine and Pathology and Hospital Dentistry Dental School, National and Kapodistrian University of Athens Athens Greece; ^38^ Department of Radiation Oncology Centre Leon Berard Lyon France; ^39^ Hermann‐Holthusen‐Institute for Radiation Oncology Asklepios Tumorzentrum Hamburg, Asklepios Hospital St. Georg Hamburg Germany; ^40^ University Cancer Centre Mainz Germany

**Keywords:** CTCAE, dysphagia, head and neck cancer, survivor, toxicity, xerostomia

## Abstract

Head and neck cancer (HNC) patients may experience toxicities as a result of their treatment modality. While acute toxicities have been well documented, the prevalence of toxicities at long‐term follow‐up of HNC survivors is less clear. As part of a multi‐national, cross‐sectional study, HNC survivors at least 5 years post‐diagnosis were invited to undergo a toxicity examination. Using the Common Terminology Criteria for Adverse Events (version 5), 33 toxicities were assessed. From 2019 to 2021, 1094 survivors from 26 sites in 11 countries completed the assessment. Eighty‐seven percent were from Europe, and most were survivors of oropharynx (35%), oral cavity (21%), or larynx cancer (19%). The majority had been diagnosed at stage III or IV (62%), and the median time since diagnosis was 8 years (range 5–36). Most had been treated with surgery and radiotherapy with or without chemotherapy (38%). Six percent had no toxicities, and 26% had only mild toxicities. 68% had at least one moderate or severe late toxicity. Overall, the most frequent late toxicities at any grade were dry mouth (67%), soft tissue fibrosis (52%), dysphagia (51%), and voice alterations (39%). Fistulae, neck and face edema, and osteonecrosis of the jaws were present in very few survivors. Our study shows that the majority of HNC survivors experience moderate or severe late toxicities, but that the problems are concentrated in a small group of specific toxicities. Understanding the problems experienced in the long term can help better inform newly diagnosed patients as well as inform survivorship follow‐up initiatives.

## What's New?

Cancer treatments frequently have toxic effects on the body, and these toxicities may cause long‐term health issues. Here, the authors report the results of a large, international study to evaluate long‐term toxicities of treatment for head and neck cancer (HNC). People with HNC who survived at least five years past diagnosis were assessed for 33 different toxicities. The majority (68%) had at least one moderate to severe symptom arising from their cancer treatment. The most common were dry mouth, soft tissue fibrosis, difficulty swallowing, and voice alterations. Awareness of these symptoms could help direct preventative and mitigating measures.

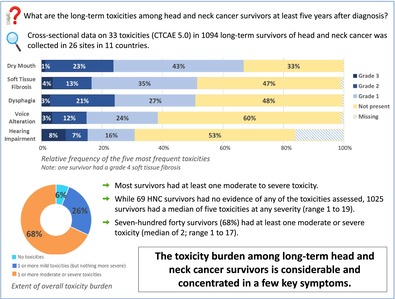

**Abbreviations**
CHES: Computer‐Based Health Evaluation System
CRT: chemoradiotherapy
CT: chemotherapy
CTCAE: Common Terminology for Adverse Events
EORTC: European Organisation for Research and Treatment of Cancer
HNC: head and neck cancer
HNCG: Head and Neck Cancer Group
HPV: human papilloma virus
IMRT: intensity modulating radiotherapy
LENT: Late Effects of Normal Tissue
QLG: Quality of Life Group
RT: radiotherapy
RTOG: Radiation Therapy Oncology Group
SAS: Statistical Analysis Software
SOMA: Subjective Objective, Management and Analytic

## 1 INTRODUCTION

According to Global Cancer Observatory data from 2022, head and neck cancer (HNC) were the sixth most commonly diagnosed cancers worldwide, with approximately 946,000 new diagnoses and 482,000 deaths in that year.^1^ Incidence within the sub‐sites varies, with oral cavity/lip and larynx being among the most frequent sites and salivary glands among the least frequent.^2^ HNC incidence is expected to increase in the coming decades, due in part to changing lifestyle factors such as tobacco and alcohol consumption in some countries,^3^, ^4^ but in particular due to an increase in human papilloma virus (HPV) prevalence, which is an increasing cause of oropharyngeal cancer.^4^, ^5^ While there is evidence of improved survival in the past decade,^6^, ^7^ examples of five‐year relative survival proportions range from about 90% for lip cancer to about 25% for hypopharyngeal cancer.^2^

Treatment recommendations for HNC vary in accordance with the sub‐site, stage, and involvement of lymph nodes and usually include surgery, radiotherapy (RT), and chemotherapy (CT), either alone or in combination.^8^ Early stage tumors can be treated with surgery or RT, while advanced disease likely requires definitive chemoradiotherapy (CRT) or surgery with adjuvant RT alone or in combination with CT. The short‐term effects of treatment are well known.^9^ As examples, 25% of 137 HNC patients (primarily oropharynx and larynx cancer) in Scotland were found to have trismus at 6 months post RT,^10^ 22% of 238 HNC patients (primarily larynx and oropharynx cancer) treated with RT or CRT in the Netherlands had grade 2–4 swallowing dysfunction at 6 months post‐treatment (according to the RTOG/EORTC Late Radiation Morbidity Scoring Criteria),^11^ 72% of 212 HNC patients within 12 weeks of treatment conclusion had dry mouth at a grade 2 severity (according to the Common Terminology for Adverse Events [CTCAE]) and 72% had dysphagia at a grade 2 or 3^12^; as well, these patients are known to suffer from hearing loss^13^ and considerable pain.^14^

However, the prevalence of toxicities among survivors who are five years or more past their diagnosis is not as well known. This is demonstrated by a recent review of studies using the CTCAE to assess toxicities in the long term^15^; three articles were identified, comprising 66,^16^ 242,^17^ and 789^18^ survivors of nasopharyngeal cancer, and the toxicities assessed were limited to a small selection of endpoints. A lack of a clear understanding of the challenges faced by this survivor group hinders the ability to provide effective support options as well as limits the ability to offer newly diagnosed patients a clear vision of what long‐term survivorship entails. This knowledge gap prompted a study on the toxicities present in long‐term HNC survivors known as the EORTC 1629 study (European Organisation for Research and Treatment of Cancer).

## 2 METHODS

The EORTC 1629 study is a multi‐national, cross‐sectional study initiated by the EORTC Quality of Life Group (QLG) and the EORTC Head and Neck Cancer Group (HNCG). At the meetings of both groups, potential study collaborators were informed about the project and the possibility to enroll long‐term survivors, defined as being at least 5 years post‐diagnosis. No restrictions were made on the type of treatment or the type of medical profession other than an assurance that it would be possible to enroll at least 10 survivors.

Study collaborators reviewed their records and contacted survivors fulfilling the inclusion criteria, which were: at least 18 years old at the time of the survey, a confirmed carcinoma of the larynx, lip, oral cavity, salivary glands, oropharynx, hypopharynx, nasopharynx, nasal cavity, paranasal sinuses, or unknown primary in the head and neck area, and the diagnosis more than 5 years in the past. Exclusion criteria were thyroid or eye tumors, skin cancer, or lymphoma. Survivors were contacted by telephone, letter, or during routine visits and were informed about the study. Survivors who agreed to participate were invited to a clinical visit, where a clinician examined them for 33 toxicities using version 5 of the CTCAE.^19^ If a study collaborator was not a medical doctor (e.g., if they were an oncological dentist), instructional training on the toxicity examination was carried out via video call with a medical oncologist before any survivors were enrolled. The clinician documented the survivor's disease and treatment history in a case report form, which included the patient's sex, age, years of education, smoking status, diagnosis and treatment details, Karnofsky index, and Charlson Comorbidity Index.^20^

The 33 specific late toxicities to be assessed in the study were selected through discussions with the collaborating clinicians at the semi‐annual meetings for the QLG and HNCG as well as email correspondence. A contact clinician in the HNCG was established, and he assisted in the coordination of toxicity selection. Clinicians were free to suggest any toxicity they felt was important to investigate. Total agreement to add a toxicity was not required. The toxicities included were dry mouth, soft tissue fibrosis (neck fibrosis), dysphagia, voice alteration, hearing impairment, hypothyroidism, aspiration, neck pain, tinnitus, peripheral sensory neuropathy, trismus, oral pain, pharyngolaryngeal pain, peripheral motor neuropathy, neck edema, face edema, brachial plexopathy, injury to carotid artery, osteonecrosis, myelitis, oral cavity fistula, and pharyngeal fistula as well as 11 cranial neuropathies (disorders of the olfactory nerve, optic nerve, oculomotor nerve, trochlear nerve, trigeminal nerve, abducens nerve, facial nerve, acoustic nerve, glossopharyngeal nerve, accessory nerve, and hypoglossal nerve).

The toxicity evaluation and the case report form were completed on paper and then sent to the coordinating center in Mainz, Germany, where the data were entered into an electronic, web‐based data capture system called Computer‐Based Health Evaluation System (CHES) created by a company based in Austria.^21^ Sites also had the option to directly enter their data into CHES by requesting a login and password to CHES from the coordinator in Mainz.

The survivor characteristics are reported as frequencies and percentages except for age, which is reported as the mean and range in years. The frequency of patients with no toxicities, only one or more grade 1 toxicities, and those with at least one grade 2 or more is reported, as is the median number of toxicities in the latter two groups. As well, the median number of toxicities experienced among all patients with at least one toxicity at any severity is reported. For each toxicity, the relative frequency is reported by severity. All analyses were done with Statistical Analysis Software (SAS), version 9.4.

## 3 RESULTS

### 3.1 Characteristics of the survivors

**FIGURE 1:**
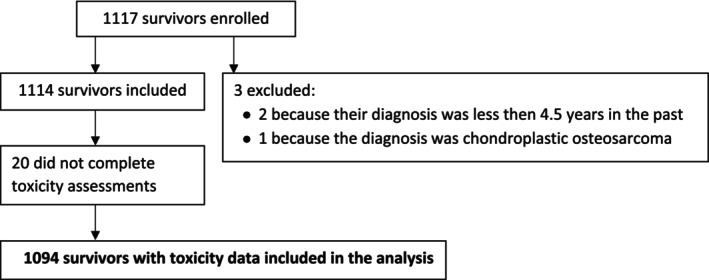
Flow of survivors enrolled in the EORTC 1629 study.

Between October 2019 and October 2021, 1117 survivors were enrolled, of whom three were excluded because of an ineligible diagnosis or being less than five years post‐diagnosis (Figure 1). A further 20 ultimately did not complete a toxicity assessment, so that 1094 survivors were included for analysis. In all, 26 sites in 11 countries enrolled survivors, with the highest enrollment in Italy, Belgium, Germany, Norway, and Brazil. The majority (71%) of the survivors were men, and the mean age was 65 years (median 66 years) (Table 1). Most of the survivors were former smokers (58%), and 42% were from Central/Western Europe. Squamous cell carcinoma (88%) was the most common histology. The largest sub‐site groups were cancer of the oropharynx (35%), oral cavity (21%), and larynx (19%). A little more than a third of the patients were diagnosed at UICC stage IV (39%), and a similar proportion had been treated with surgery and radiotherapy (38%). Nearly a quarter of survivors (24%) had been treated with monotherapy. Among survivors who had received RT (monotherapy or multimodal), 54% had received IMRT and 34% 3D‐conformal.

**TABLE 1:** Characteristics of the 1094 survivors at inclusion in the study.

	*n*	%
Totals	1094	100
Sex		
Male	779	71.2
Female	315	28.8
Age (years)		
Mean (range)	65 (23–93)	
Geographic area		
Northern Europe	251	22.9
Central /Western Europe	458	41.9
Southern Europe	238	21.8
Israel	9	0.8
Japan	30	2.7
Brazil	108	9.9
Smoking status		
Never smoker	317	29.0
Former smoker	637	58.2
Current smoker	139	12.7
Missing	1	0.1
Total years of education		
<10	378	34.6
10	163	14.9
>10	542	49.5
Missing	11	1.0
Tumor subsite		
Oropharynx	378	34.6
Oral cavity	231	21.1
Larynx	206	18.8
Nasopharynx	84	7.7
Hypopharynx	48	4.4
Salivary glands	59	5.4
Nasal cavity and sinuses	37	3.4
Unknown primary	51	4.7
Histology		
Squamous cell	960	87.8
Other	124	11.3
Missing	10	0.9
UICC stage		
I	216	19.7
II	173	15.8
III	247	22.6
IV	426	38.9
Missing	32	2.9
Treatment		
Surgery	124	11.3
RT	134	12.2
CRT	311	28.4
RT ± CT and ND	109	10.0
Surgery and RT ± CT	415	37.9
Missing	1	0.1
Karnofsky index		
90 or 100	740	67.6
70 or 80	311	28.4
50 or 60	35	3.2
Less than 50	3	0.3
Missing	5	0.5
Charlson comorbidity index		
0	693	63.3
1	206	18.8
2	92	8.4
≥3	103	9.4
Current evidence of disease		
Yes	32	2.9
No	1060	96.9
Missing	2	0.2
Second primary		
Yes	159	14.5
No	928	84.8
Missing	7	0.6
Time since diagnosis (years)		
5–6	243	22.2
7–8	381	34.8
9–10	215	19.7
>10	255	23.3

*Note:* Oropharynx includes base of tongue and tonsil. In the RT ± CT and ND group: there are 33 (30%) with no CT. In the Surgery and RT ± CT: there are 229 (55%) with no CT.

Abbreviations: CT, chemotherapy; CRT, chemo‐radiotherapy; ND, neck dissection; RT, radiotherapy; UICC, Union for International Cancer Control.

In our study, most survivors had not developed a second primary (85%), did not have current evidence of disease (97%), and had high functioning according to the Karnofsky index (68% had a score of 90 or 100) at the time of clinical examination. The time since diagnosis ranged from 5 to 36 years. The median time since diagnosis was 8 years.

### 3.2 Toxicity frequency

Across all toxicities, 69 (6%) of the HNC survivors had no evidence of any of the toxicities assessed, while 1025 survivors had a median of five toxicities (range 1–19). Two hundred and eighty‐five survivors (26%) had one or more grade 1 toxicities but no toxicities at a higher grade. Among the 285 survivors with only one or more grade 1 toxicities, 85 had only one toxicity, and the median number of toxicities was 2 (range 1–9). Seven hundred and forty survivors (68%) had at least one toxicity at grade 2, 3, or 4, with a median of 2 toxicities (range 1–17) at a grade 2 or higher. Six survivors had one grade 4 toxicity: one related to a carotid artery injury that had been taken care of according to clinical guidelines, one related to soft tissue fibrosis, two related to optic nerve damage, and two to aspiration. The two patients with severe aspiration had had laryngeal closure with tracheostomies that were required to mitigate this.

**FIGURE 2:**
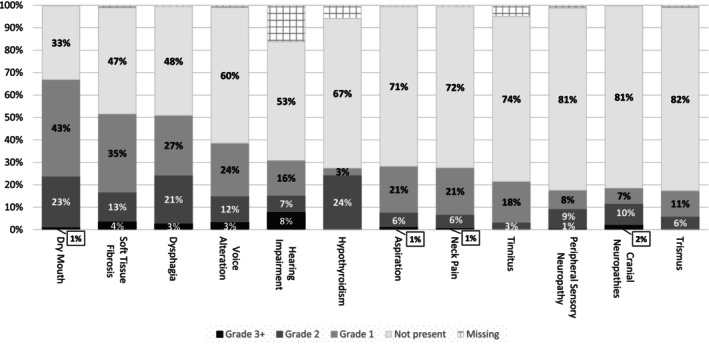
Severity distribution of investigated toxicities (1 of 2). Total *n* for each toxicity is 1094. Grades are according to the Common Terminology Criteria for Adverse Events (CTCAE; Version 5).

The top five toxicities in terms of frequency among all 1094 survivors were dry mouth (*n* = 732, 67%), soft tissue fibrosis (*n* = 564, 52%), dysphagia (*n* = 557, 51%), and voice alterations (*n* = 422, 39%); hearing impairment was the fifth most common (*n* = 338, 31%), but 178 survivors had missing values here, so it is possible the percentage was higher (Figure 2). If only survivors with at least one toxicity at grade 2 or higher were considered (*n* = 740), these five toxicities were still among the most frequent (dry mouth: *n* = 261, 24%; soft tissue fibrosis: *n* = 186, 17%; dysphagia: *n* = 266, 24%; voice alteration: *n* = 164, 15%; hearing impairment: *n* = 168, 15%), but hypothyroidism (*n* = 268, 24%) was also notable. Hearing impairment and hypothyroidism had respectively 16% and 6% missings, so it is possible both were higher. Among the 528 survivors who had received both RT and CT treatment, 97 (18%) had grade 2 or grade 3 hearing impairment, which was more than the proportion of survivors who had had RT but no CT (*n* = 386, 10%); however, 20 of the 123 survivors (16%) with neither RT nor CT also had moderate to severe problems with hearing. For hypothyroidism, there were 266 survivors with grade 2 severity and 1 with grade 3. When looked at by age and sex, females had a greater proportion of grade 2 or 3 hypothyroidism than men across three age groups ([males aged ≤60: 47/197 (24%); aged 61–75: 90/456 (20%); aged >75: 25/126 (20%)] [females aged ≤60: 30/99 (30%); aged 61–75: 64/169 (38%); aged >75: 11/47 (23%)]).

Aspiration can have immediate consequences for a patient, and in our survival sample was a problem at any grade in 309 survivors (28%), whereby 14 (1%) had grade 3 or 4. We observed that among the 124 patients who only received surgery, 10% had grade 1 aspiration, none had grade 2, and 1 survivor had grade 3, whereas 5% of the 134 survivors treated with RT, 9% of the 311 survivors treated with CRT, 16% of the 109 survivors treated with RT ± CT and neck dissection, and 7% of the 415 survivors treated with surgery and RT ± CT had grade 2/3 aspiration (one of the RT ± CT and neck dissection had a grade 4).

**FIGURE 3:**
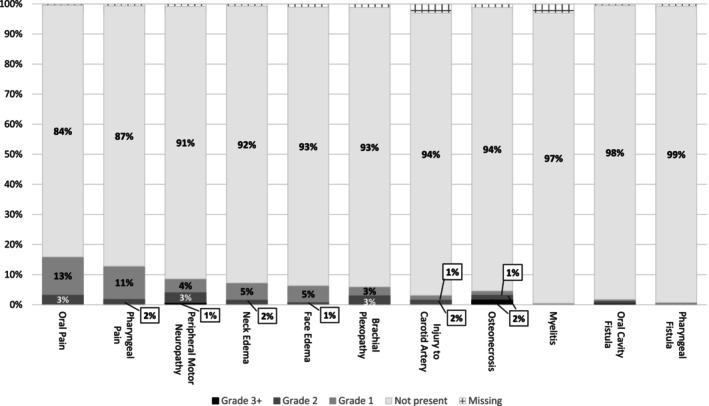
Severity distribution of investigated toxicities (2 of 2). Total *n* for each toxicity is 1094. Grades are according to the Common Terminology Criteria for Adverse Events (CTCAE; Version 5).

Among the five least frequent toxicities observed at any grade were 50 survivors with osteonecrosis (5%) (47 of whom had had multimodal treatment), 34 survivors with a carotid artery injury (3%), 19 survivors with an oral cavity fistula (2%), eight survivors with a pharyngeal fistula (1%), and 5 with myelitis (<1%) (Figure 3). Neck and face edema at any severity was observed in 79 (7%) and 69 (6%) of all survivors, respectively. Considering the pain toxicities, neck pain was more frequent in the 1094 survivors (*n* = 302; 28%) than oral pain (*n* = 174; 16%) and pharyngeal pain (*n* = 140; 12%). Peripheral sensory neuropathy was observed more often than peripheral motor neuropathy (*n* = 192; 18% versus *n* = 94; 9%), but both had about 1% with grade 3. Survivors who had had CT had a greater proportion of moderate to severe problems with peripheral sensory neuropathy (13%) compared to those without CT (6%). Of the 46 patients who experienced moderate to severe motor neuropathy, only 11 also had moderate to severe accessory nerve disorder.

Two‐hundred and three (19%) survivors had at least one cranial neuropathy of any severity, and 128 (12%) had at least one with a grade 2 or more. Of the 203 with at least one cranial neuropathy, 62 (31%) had an accessory nerve disorder, 59 (29%) an acoustic nerve disorder, 54 (27%) an olfactory nerve disorder, 51 (25%) a glossopharyngeal nerve disorder, and 44 (22%) a hypoglossal nerve disorder. The remainder of the cranial neuropathies were less frequent, in particular disorders of the trochlear nerve and abducens nerve, of which none were reported.

## 4 DISCUSSION

To our knowledge, this is the largest study on the prevalence of late toxicities in long‐term survivors of head and neck cancer to date. Our findings provide evidence that long after treatment, when most individuals are cured of their cancer, most survivors experience some sort of late toxicity. In particular, dry mouth, soft tissue fibrosis, dysphagia, voice alteration, hearing impairment, and hypothyroidism are highly prevalent, whereas other problems such as fistula, edema, myelitis, and osteonecrosis of the jaws were seldom observed. We found that 68% had at least one moderate (grade 2) or severe (grade 3 or higher) late toxicity that might affect their functioning in daily life. Of particular note is aspiration, which is a dangerous toxicity that can be fatal^22^; it is therefore concerning that 7% of the survivors in our study had moderate or severe problems with this. The 43% of survivors in our study with grade 1 dry mouth may not have had to alter their eating habits, but adequate salivary production is also important for wound healing in the oral cavity, facilitating speech, and tooth mineralization,^23^, ^24^ showing how interdependent some toxicities are.

Published results on late toxicity occurrence among long‐term survivors are rare, as indicated by a literature review identifying only three studies that used the CTCAE as the assessment instrument.^15^ The studies that are available generally base conclusions on a considerably smaller number of survivors.^17^, ^25^ In a Belgian study of 152 HNC survivors diagnosed 5 years in the past, similar results to ours were found; at any severity, 72% had dry mouth, 65% had soft tissue fibrosis, and 40% had dysphagia using the Late Effects of Normal Tissue (LENT)—Subjective Objective, Management and Analytic (SOMA) toxicity classification system.^25^ All patients had had RT either alone or with surgery or CT. Our results of 24% having dysphagia at CTCAE grade 2 or higher are also similar to the 21% found in 239 HNC survivors at 5 years post diagnosis in Norway.^26^ Our finding that 24% had moderate to severe hypothyroidism shows a considerably larger proportion than a recent review on prevalence in a range of populations, including non‐cancer populations, with up to 5.3% in Europe depending on the specific population and definition of the condition used.^27^ While very few survivors had a grade 4 toxicity, the implications for these individuals is considerable. Carotid artery injury is a life‐threatening condition that requires immediate surgical intervention. Unfortunately, optic nerve damage and soft tissue fibrosis cannot be regenerated, and the patient will need time to adjust. Severe fibrosis leading to impaired feeding may be managed by surgical blocking.

However, as expected due to the disease site, a considerably higher proportion of survivors with moderate to severe toxicity (CTCAE grade ≥2) were reported by Tsai et al. in a Taiwanese group of 242 long‐term nasopharyngeal survivors treated between 1998 and 2007 who had received RT.^17^ Hearing loss was reported for 51%, dysphagia for 41%, dry mouth for 56%, and neck fibrosis for 27%. The older RT technology during part of that time (only 41% had intensity modulating radiotherapy [IMRT]) could be an explanation for this in addition to the extensive treatment these patients received. Although nasopharyngeal cancer is a HNC, these patients are not directly comparable to our study of mixed HNCs. The explanation for the considerably higher proportion of hearing loss in the study by Tsai et al. is likely the extent of RT the patients received, which may have included the inner ear. Cisplatin has known ototoxic effects,^28^ which also could be an explanation, as cisplatin has a prominent role in treating advanced, non‐metastatic HNC,^8^ and 66% of the patients reported by Tsai had had CT. Our hearing impairment results contain 16% missing data, introducing uncertainty in the true extent of this problem in our study. Hearing was asked about in two sections: one for participants in a monitoring program for hearing impairment and one for participants not enrolled in such a program, and this caused confusion. Our finding that peripheral sensory neuropathy was more common in survivors who had received chemotherapy was not surprising. Cisplatin is the most frequently used agent in curative concomitant chemoradiotherapy for HNC^29^, ^30^ and can induce peripheral neuropathy. Unfortunately, there is no preventive treatment available.^28^ There was not a notable overlap between moderate/severe peripheral motor neuropathy and moderate/severe accessory nerve disorder. Injury to the accessory nerve is a known complication after neck surgery that sometimes cannot be avoided if the tumor grows close to or around the nerve. However, there is a high awareness among the surgeons regarding this complication.^31^

A Chinese study from 2014 including 789 nasopharyngeal cancer patients and survivors after IMRT is a notable exception to the small samples reported in the literature, but the follow‐up time was very variable and ranged from 4 to 106 months, and the limitations of comparing to a sample of nasopharynx cancer survivors persist.^18^ The Chinese authors reported a slightly smaller proportion of patients (56%) with one or more moderate or severe toxicities than our study.

Our results show that long‐term survivors of HNC have a continued need for supportive care in managing the effects of treatment. As an example, the high prevalence of dry mouth has ongoing implications for oral health, requiring meticulous oral hygiene in order to avoid severe dental issues^32^ as well as the functional limitations for sleep quality and swallowing.^33^ More than half the survivors in our study had mild dysphagia or worse, showing a need for ongoing support specifically for this. The additional effect of aging over time could contribute to worsening dysphagia^34^ in this already vulnerable population. The most frequent toxicities identified in our study have implications beyond immediate consequences, including associations with increased sticky saliva, decreased sleep quality, physical functioning,^35^ reducing quality of life. Survivorship care should include monitoring these problems and referral for supportive intervention where possible.^36^

## 5 LIMITATIONS

Our study likely suffers from healthy survivor bias in that survivors who are doing well may be more likely to have agreed to participate, in particular because the study required a clinical visit. Therefore, it is possible that the toxicity burden in HNC survivors who are five or more years post‐diagnosis may be higher than our findings show. Alternatively, it is also possible that survivors experiencing problems were more motivated to attend a clinical visit than those who were having no problems. We lack information on the human papilloma virus status of these patients, the presence of which is a known positive prognostic indicator.^37^ However, given the inclusion criterion that the survivors were at least 5 years post‐diagnosis, we think it unlikely that many survivors would have had this information in their medical record. The long‐term implications of HPV‐related disease will be an important aspect to consider for future studies. Unfortunately, we were not able to collect data on non‐responders due to data protection regulations in some countries and therefore cannot comment on characteristics that non‐responders might share.

## 6 STRENGTHS

Among the few studies in the literature that examine toxicities among HNC long‐term survivors, the range of toxicities assessed is quite narrow; the publications cited in the previous paragraph often only reported the toxicities specified, whereas our study had a wide range of toxicities, which we consider a strength in our work in addition to the large multi‐national sample of HNC survivors. Our results concerning the toxicities that were not present for most survivors are also valuable, as supportive care resources are limited and should be directed to benefit the greatest number of survivors. Understanding what the more frequent problems are for long‐term survivors can be useful to inform newly diagnosed patients about what problems may be anticipated in the long‐term and can contribute to establishing long‐term follow‐up routines to assess the toxicities that are more likely to occur.

## AUTHOR CONTRIBUTIONS

**Katherine J. Taylor:** Conceptualization; methodology; formal analysis; data curation; writing – original draft; writing – review and editing; investigation. **Cecilie D. Amdal:** Investigation; conceptualization; methodology; writing – review and editing. **Kristin Bjordal:** Conceptualization; methodology; investigation; writing – review and editing. **Guro L. Astrup:** Conceptualization; methodology; investigation; writing – review and editing. **Bente B. Herlofson:** Conceptualization; methodology; investigation; writing – review and editing. **Fréderic Duprez:** Conceptualization; methodology; investigation; writing – review and editing. **Ricardo R. Gama:** Conceptualization; methodology; investigation; writing – review and editing. **Alexandre Jacinto:** Conceptualization; methodology; investigation; writing – review and editing. **Eva Hammerlid:** Conceptualization; methodology; investigation; writing – review and editing. **Melissa Scricciolo:** Investigation; writing – review and editing. **Femke Jansen:** Conceptualization; methodology; investigation; writing – review and editing. **Irma M. Verdonck‐de Leeuw:** Conceptualization; methodology; investigation; writing – review and editing. **Giuseppe Fanetti:** Investigation; writing – review and editing. **Orlando Guntinas‐Lichius:** Conceptualization; methodology; investigation; writing – review and editing. **Johanna Inhestern:** Conceptualization; methodology; investigation; writing – review and editing. **Tatiana Dragan:** Investigation; writing – review and editing. **Alexander Fabian:** Investigation; writing – review and editing; methodology. **Andreas Boehm:** Conceptualization; investigation; methodology; writing – review and editing. **Ulrike Wöhner:** Conceptualization; methodology; investigation; writing – review and editing. **Naomi Kiyota:** Conceptualization; methodology; investigation; writing – review and editing. **Maximilian Krüger:** Investigation; writing – review and editing. **Pierluigi Bonomo:** Conceptualization; methodology; investigation; writing – review and editing. **Monica Pinto:** Conceptualization; investigation; methodology; writing – review and editing. **Sandra Nuyts:** Investigation; writing – review and editing. **Joaquim Castro Silva:** Investigation; writing – review and editing; conceptualization; methodology. **Carmen Stromberger:** Investigation; writing – review and editing. **Pol Specenier:** Investigation; writing – review and editing. **Francesco Tramacere:** Funding acquisition; writing – review and editing. **Ayman Bushnak:** Investigation; writing – review and editing. **Pietro Perotti:** Investigation; writing – review and editing. **Michaela Plath:** Investigation; writing – review and editing. **Alberto Paderno:** Investigation; writing – review and editing. **Noa Stempler:** Investigation; writing – review and editing. **Maria Kouri:** Investigation; conceptualization; methodology; writing – review and editing. **Vincent Grégoire:** Supervision. **Silke Tribius:** Writing – review and editing; methodology. **Susanne Singer:** Conceptualization; methodology; writing – review and editing; funding acquisition; supervision.

## FUNDING INFORMATION

This work was funded by the EORTC Quality of Life Group (EORTC 1629). The EORTC Quality of Life Group business model involves charges for commercial companies using EORTC instruments. Academic use of EORTC instruments is free of charge.

## CONFLICT OF INTEREST STATEMENT

S. Singer has received consulting fees from Lilly that were outside of this study. A. Fabian has received honoraria from Merck Sharp and Dohme outside the field of this work. M. Pinto has received consulting fees from Meeting & Words S.r.l. and Hinovia S.r.l., a speaker fee from Becton Dickinson Italia S.p.A, and has participated as a co‐investigator in a study funded by Amgen, all of which are outside of this study. All other authors report no conflicts of interest.

## ETHICS STATEMENT

All participants gave written informed consent before enrollment in the study, which was conducted in accordance with the principles of the Declaration of Helsinki. The ethical approval at the coordinating center in Mainz, Germany, was granted by the Landesärztekammer (Medical Association) Rhineland‐Palatinate (No. 2018‐13579) and was obtained at each site in accordance with local regulations.

## Data Availability

Data may be requested from the data repository of the EORTC (https://www.eortc.org/data-sharing/). Further information is available from the corresponding author upon request.
